# Clinical practice guideline for the prevention, early detection, diagnosis, management and follow up of type 2 diabetes mellitus in adults

**Published:** 2016-06-30

**Authors:** Pablo M Aschner, Oscar Mauricio Muñoz, Diana Girón, Olga Milena García, Daniel Gerardo Fernández-Ávila, Luz Ángela Casas, Luisa Fernanda Bohórquez, Clara María Arango T, Liliana Carvajal, Doris Amanda Ramírez, Juan Guillermo Sarmiento, Cristian Alejandro Colon, Néstor Fabián Correa G, Pilar Alarcón R, Álvaro Andrés Bustamante S

**Affiliations:** 1 Hospital Universitario San Ignacio, Bogota, Colombia; 2 Pontificia Universidad Javeriana, Bogota, Colombia; 3 Asociación Colombiana de Diabetes, Bogota, Colombia; 4 Departamento de Epidemiología y Bioestadística. Pontificia Universidad Javeriana, Bogota, Colombia; 5 Asociación Colombiana de Endocrinología, Universidad del Valle, Cali, Colombia; 6 Federación diabetológica Colombiana, Bogota, Colombia; 7 Universidad Nacional de Colombia, Bogota, Colombia; 8 Universidad de Antioquia, Medellin, Colombia

**Keywords:** Type 2 diabetes mellitus, clinical practice guidelines

## Abstract

In Colombia, diabetes mellitus is a public health program for those responsible for creating and implementing strategies for prevention, diagnosis, treatment, and follow-up that are applicable at all care levels, with the objective of establishing early and sustained control of diabetes. A clinical practice guide has been developed following the broad outline of the methodological guide from the Ministry of Health and Social Welfare, with the aim of systematically gathering scientific evidence and formulating recommendations using the GRADE (Grading of Recommendations Assessment, Development and Evaluation) methodology. The current document presents in summary form the results of this process, including the recommendations and the considerations taken into account in formulating them. In general terms, what is proposed here is a screening process using the Finnish Diabetes Risk Score questionnaire adapted to the Colombian population, which enables early diagnosis of the illness, and an algorithm for determining initial treatment that can be generalized to most patients with diabetes mellitus type 2 and that is simple to apply in a primary care context. In addition, several recommendations have been made to scale up pharmacological treatment in those patients that do not achieve the objectives or fail to maintain them during initial treatment. These recommendations also take into account the evolution of weight and the individualization of glycemic control goals for special populations. Finally, recommendations have been made for opportune detection of micro- and macrovascular complications of diabetes.

## Abstract (es)

En Colombia la Diabetes Mellitus es un problema de salud pública por lo que deben generarse e implementarse estrategias de prevención, diagnóstico, tratamiento y seguimiento, aplicables en todos los niveles de atención con miras a establecer el control de la diabetes en forma temprana y sostenida. Se elaboró una guía de práctica clínica siguiendo los lineamientos de la guía metodológica del Ministerio de Salud y Protección Social para recolectar de forma sistemática la evidencia científica y formular las recomendaciones utilizando la metodología GRADE. El presente documento muestra, de forma resumida, el resultado de ese proceso, incluyendo las recomendaciones y las consideraciones tenidas en cuenta para llegar a ellas. En términos generales, se propone un proceso de tamización mediante el cuestionario FINDRISC adaptado a población Colombiana que permite llegar a un diagnóstico temprano de la enfermedad y un algoritmo para el manejo inicial que es generalizable a la gran mayoría de los pacientes con diabetes mellitus tipo 2 y que es sencillo de aplicar en atención primaria. También se hacen unas recomendaciones para escalar el tratamiento farmacológico de los pacientes que no alcanzan la meta o la pierden con el manejo inicial, teniendo en cuenta principalmente la evolución del peso y la individualización de la meta de control glucémico en poblaciones especiales. Finalmente se proponen algunas recomendaciones para la detección oportuna de las complicaciones micro y macrovasculares de la diabetes.

## Introduction

In 2006, the General Assembly of the United Nations approved a historic resolution that recognized the global threat of the diabetes epidemic. For the first time, governments recognized that a non-infectious disease poses a threat to world health of equal gravity to that posed by infectious diseases such as AIDS, tuberculosis, or malaria. In Colombia, between 7 and 9 percent of the adult population (20 years and over) have diabetes mellitus type 2 (DMT2), with a prevalence five times less in rural areas 1. Based on data compiled from the most recent edition of the Atlas of the International Diabetes Federation 2, it has been calculated that in Colombia, more than 2 million people have diabetes, and the vast majority of these have type 2. About 50% of these people are not aware of their condition. In Colombia, diabetes mellitus is one of the five largest causes of death and one of the 10 major reasons that adults seek medical help. Consequently, this disease constitutes a public health problem that must be managed at all care levels, with prevention strategies for every stage, to establish early and sustained control of diabetes.

Diabetes control must be early, effective, and sustained to prevent chronic complications and avoid the deleterious effect of metabolic memory 3. Experts suggest the use of a glycosylated hemoglobin level (HbA1c) of 6.5% to diagnose diabetes and to take this value as a starting point for its management. Controlled and randomized clinical studies like The United Kingdom Prospective Diabetes Study (UKPDS) 4,5 have shown that if DMT2 is adequately treated from its inception, the incidence of chronic complications attributable to prolonged hyperglycemia can be reduced. In particular, damage to the retina (retinopathy), the kidneys (nephropathy), and the peripheral nervous system (neuropathy) at the end of 10 years was reduced by an intensive management strategy using insulin and sulfonylureas, and the incidence of cardiac infarctions and death was reduced using metformin. With longer follow-up over 20 years, a reduction in fatal and nonfatal cardiovascular events was observed with all the antidiabetic medications mentioned 5. HbA1c, the measurement associated with these benefits, remained at an average of 7% and must be used as the objective of glycemic control. Treatment must be multifactorial because this is the most effective strategy in the medium and long term for controlling all cardiovascular risk factors including high blood sugar, lipids, and blood pressure 6,7.

Various studies have demonstrated that intervention with those at high risk of developing diabetes can delay its appearance, and various countries are implementing screening strategies to this end (Finland, for example). Such an intervention can also identify subjects with unrecognized diabetes who can benefit from treatment that reduces the incidence of complications 2. Risk scales already exist to facilitate such screening (for example, FINDRISC) 8.

Diabetes management is far from simple; in fact, every day doctors are faced with more and more drugs and devices, all with proven efficacy and safety, which put them in a dilemma regarding when and how to prescribe them and to which patients. In most cases, the primary care doctor must make these decisions. Moreover, because diabetes is a chronic and progressive illness that can lead to complications, the physician must work with a multidisciplinary team that will ensure patient education and compliance and must know when and how to seek the assistance of specialists. The current trend towards individualized diabetes management goals and treatments is not compatible with the large volume of patients that the primary care physician must see and the limited time available to attend to them. The World Health Organization (WHO) has proposed the establishment of teams made up of a physician trained in diabetes and a diabetes educator at the primary care level, where most patients with uncomplicated DMT2 must be dealt with. Every patient with DMT2 must have access to a structured lifestyle change program that helps the patient achieve and maintain a body mass index in the appropriate range, a physical activity routine, and control of cardiovascular risk factors, including the glycemic index 9.

The Ministry of Health and Social Welfare has mandated the Pontificia Universidad Javeriana and the Alianza de las Universidades Javeriana, Nacional y Antioquia (Alianza CINETS) to develop a clinical guide on the Diagnosis, Treatment, and Follow-Up of Diabetes Type 2 in the Population 18 Years and Over. The present document presents recommendations for good practice that are based on the best available clinical evidence and on additional relevant considerations for government programs such as cost, patient preferences, and the benefit-risk relationship for the technologies of interest.

### Objectives, scope, and target population of the guide

This document does not claim to present an extensive compilation of clinical information. On the contrary, its objective is to provide clear, concise, and simple responses to specific questions that were selected for their clinical importance, giving priority to topics believed to be already subjects of debate among researchers and clinicians. Topics were also included where there was evidence of unexplained variability in medical practice by health professionals in Colombia. Statements that are widely accepted in the scientific literature worldwide and therefore do not merit a new analysis of the literature will be accepted as assumptions in the present guide and presented as such in this document.

**General objective. **To provide guidelines for clinical practice based on the best available evidence for health care and for rational use of resources in the diagnosis, treatment, and follow-up of DMT2 in the population 18 years and over. 

**Specific objectives. **

• To determine the usefulness of screening strategies as a tool for early detection and diagnosis of DMT2 in the target environment.

• To indicate the appropriate pharmacological interventions in the context of a multifactorial treatment plan, graduated from lesser to greater complexity and emphasizing effectiveness and safety, and with goals applicable to most patients with DMT2 in primary care.

• To define strategies for early detection of renal and cardiovascular complications in patients with DMT2.

**Target population. **

The recommendations presented here are targeted to adults 18 years and over who are at risk for developing DMT2 or who have an established diagnosis of DMT2.

The following lists define the topics to be included or not included in the Guide.

This Guide will NOT consider the following topics:

• Treatment of patients with a high risk of developing diabetes during the diagnostic process, for example, those with abnormal fasting glycemic index and/or glucose intolerance or who have been identified as prediabetic.

• Specific treatment of complications in patients with a diagnosis of diabetes mellitus, for example, stage IV or V chronic renal disease.

• Specific treatment of obesity in the context of diabetes mellitus, with the understanding that although obesity treatment is fundamental to diabetes management, it will be addressed in a separate guide.

**Care environment and study population.** Given the importance of this disease, the recommendations of the clinical practice guide must be directed to all clinical healthcare personnel who are responsible for early detection, overall care, and follow-up of DMT2 in adults at all levels of care. This group includes: 

• General practitioners, family doctors, internists, and in general all medical personnel responsible for screening, initial treatment, or referral of patients with DMT2 

• Physicians specialized in endocrinology and diabetes or who have received formal training in diabetes.

• Healthcare personnel who work in primary care clinics for diabetes, as well as those working in diabetes education programs.

• Specialists in general and clinical nutrition.

• Staff of insurance companies and healthcare service providers involved in their programs for health promotion and preventative healthcare, screening, and oversight of primary and secondary prevention programs for cardiac and cerebrovascular conditions.

• Patients with DMT2 or with abnormal glycemic levels. 

## Materials and Methods

This clinical practice guide was developed by a multidisciplinary group of healthcare professionals with knowledge and experience in various areas (internists with various subspecialties, endocrinologists, general practitioners, family doctors, nutritionists, nurses, psychologists, and diabetes educators) as well as patient representatives. All participants in the panel presented an open declaration of their conflicts of interest. These documents are available in the complete version of the guide, which can be found (in Spanish) at: (http://gpc.minsalud.gov.co/guias/Documents/diabetes/diabetes_tipo_2_completa.pdf).

The process of developing the guide is described in detail in the manual for developers of clinical practice guides and implementation of this methodology (available in Spanish at: http://gpc.minsalud.gov.co/recursos/Documents/Guía%20Metodológica_Web.pdf). This manual consists of two basic components; the first is technical, based on analysis of the best available evidence, whereas the second is participative in the sense that multiple groups of interested experts and organizations contributed to its preparation.

It must be emphasized that the methodology used guaranteed a systematic search of the scientific evidence (including many systematic reviews of the literature as primary sources). In this way, a transparent methodology was established to select the best evidence to use and to perform a careful evaluation of its quality. The complete version of the Guide, which includes the results of all these evaluations, can be accessed (in spanish) at: http://gpc.minsalud.gov.co/Pages/Default.aspx. The methodology group prepared a summary of the available evidence and presented it to the whole panel during the recommendation generation meetings.

The quality of the whole body of evidence that served as a basis for formulating the recommendations was evaluated using the GRADE (Grading of Recommendations Assessment, Development and Evaluation) system, which was applied to the systematic reviews, randomized clinical trials, and empirical studies that were included. In the GRADE system, an evidence quality was assigned to each outcome as a criterion for qualifying later on the body of evidence for each study comparison.

The quality of a piece of evidence depends on the following factors: risk of bias, inconsistency, whether direct or indirect evidence is used, imprecision, and risk of publication bias, as well as the magnitude of the effect, the presence of a dose-response curve, and the action of potential residual confounding factors.

To present the quality of the evidence and a summary of the findings, GRADE evidence profiles were used, which were produced using the GRADEpro program with the Guideline Development Tool. The possible qualification levels of a piece of evidence, each with its meaning and graphic representation, are presented in Table 1. 

**Table 1.** Significance and graphic representation of the levels of evidence (GRADE)

Quality of evidence	Definition	Graphic representation
High	There is great confidence that the true effect is close to the estimated effect	ⴲⴲⴲⴲ
Moderate	There is moderate confidence in the estimate of the effect: it is probable that the true effect is close to the estimate, but the possibility exists of its being substantially different	ⴲⴲⴲⴱ
Low	Confidence in the estimate of the effect is limited: the true effect may be substantially different from the estimate	ⴲⴲⴱⴱ
Very low	Confidence in the estimate of the effect is very low: it is probable that the true effect is substantially different from the estimate	ⴲⴱⴱⴱ

Source: Methodological Guide for the development of Clinical Practice Guides with Economic Evaluation in the General Social Healthcare System of Colombia 10

The strength assigned to each recommendation according to the GRADE system (Table 1) is based both on the quality of the underlying evidence and on the information and judgments provided by patients and experts concerning the risk-benefit profile of the recommended alternatives, the degree to which the recommendations agree with the values and preferences of patients, the local availability and applicability of recommended technologies or alternatives, and the resource utilization and cost associated with implementing each recommendation.

The final version of the Guide was evaluated by an international peer-review group selected by the Ministry of Health and Social Protection. This group included experts both in diabetes and in methodology. Their contributions were taken into account by the group developing the guide.

The form in which the recommendations for doctors and patients should be interpreted is presented in Table 2.

**Table 2.** Implications of the two recommendation strengths according to the GRADE system.

User group	Strong recommendation	Weak recommendation
Patients	Most people in their situation will want to follow the recommended course of action, and only a small proportion will not want to do so	A substantial number of people in their situation will want to follow the recommended course of action, but many will not want to do so
Clinical specialists	Most patients should accept the recommended course of action	It is recognized that different options may be appropriate for different patients, and an additional effort should be made to help the patient make treatment decisions consistent with his or her own values and preferences; decision-making and shared decision tools could be particularly useful
Policy-makers	The recommendation could be adopted as policy in most situations	Formulation of policies requires debate and participation by various interested parties

Source: from the GRADE Profiler manual (available on the following Web page: www.who.int/hiv/topics/mtct/grade_handbook.pdf)

## Recommendations

### Topic 1. Screening and diagnosis for diabetes mellitus type 2

**Assumptions.** DMT2 can be diagnosed with any one of the following criteria:

• Fasting plasma glycemic index ≥ 126 mg/dL

• Plasma glycemic index two hours after taking 75 g of anhydrous glucose dissolved in water ≥200 mg/dL. This is an oral glucose tolerance test (OGTT), in which two measurements are taken: one basal measurement, and the second two hours after taking the dose.

• HbA1c ≥6.5% at any time.

• In the presence of symptoms (polyuria, polydipsia, and weight loss), a random plasma glycemic test ≥200 mg/dL is sufficient to establish a diagnosis. 

**Clinical question 1.¿Should the FINDRISC tool be considered an appropriate screening tool for undiagnosed diabetes in the adult population in Colombia?. **

**Answer to question 1. **The ideal approach would be a study in which one group is screened using the FINDRISC test, followed by confirmatory blood tests in accordance with the results, after which treatment would be given to those with positive blood tests. The results from this group would be compared with those from a second group that receives the screening test and a blood test at the same time, after which those with positive results receive treatment. A third group should be included that does not undergo screening, but in which treatment is given only to those identified in a clinical context. 

Unfortunately, no study of this kind exists, and therefore initially information is presented from studies comparing screening by blood tests with detection by clinical evaluation. Next, information is presented from a comparison of blood-test screening with screening based on the FINDRISC test, and finally the potential effect of these screenings on the results is approximated by critical outcomes in patients, assuming that those who test positive in the screening receive treatment.

A study carried out in the United Kingdom 11 compared three groups selected from a population cohort of subjects between 40 and 65 years of age. In the first step (1990-1992), one-third of the population chosen at random was invited to participate in screening with an OGTT and with documentation of cardiovascular risk factors. The same procedure was followed 10 years later in a second step (2000-2003) with half those who were not selected initially and who were still alive. The third group was never screened. The results of the tests were communicated to general practitioners for them to take whatever measures they considered appropriate.

 In the first stage, the subjects that responded to the invitation to participate in screening had lower mortality after 10 years of follow-up compared to those who were not invited (HR= 0.54; CI 95%= 0.40-0.74), whereas those that were invited but did not respond had higher mortality in the same period (HR= 1.36; CI 95% = 1.01-1.82 vs. uninvited). In the second stage, after 8 years of follow-up, those who accepted screening had lower mortality compared to those who were not invited (HR= 0.52; CI 95%= 0.35-0.78), whereas those who were invited but did not respond had the highest mortality of all (HR= 1.73; CI 95%= 1.34-2.24 vs. uninvited). This study leads to the conclusion that simply inviting people to take part in diabetes screening is not sufficient, but that screening must be done effectively to achieve a long-term reduction in mortality.

The FINDRISC test was constructed on the basis of data from a Finnish population cohort of subjects between 35 and 64 years of age selected at random in 1987 and followed for 10 years, with the objective of predicting development of diabetes requiring treatment with medication. It was validated with another independent cohort selected at random in 1992 and followed for 5 years 12. The FINDRISC point scale runs from 0 to 20 points, and a value greater than or equal to 9 predicted diabetes with a sensitivity of 0.78 and 0.81, a specificity of 0.77 and 0.76, and a positive predictive value of 0.13 and 0.05 in the 1987 and 1992 cohorts respectively. 

FINDRISC has also been validated in other populations. In Bulgaria, subjects were selected with at least one major risk factor for diabetes: a FINDRISC score greater than or equal to 12 had a sensitivity of 0.78 (CI 95%= 0.73-0.85) and a specificity of 0.62 (CI 95%= 0.58-0.68) for identifying subjects with diabetes or pre-diabetes 3. In Greece, the test was validated in a sample population, and a score greater than or equal to 15 had a sensitivity of 0.82 and a specificity of 0.60 for predicting the presence of undiagnosed diabetes. The area underneath the receiver operating characteristic (ROC) curve was 0.72 for detecting diabetes and 0.72 for some degree of glycemic abnormality 13. The test was also validated in Spain, where the area underneath the ROC curve was 0.74 for detecting undiagnosed DMT2 and 0.75 for predicting incipient DMT2. The score having the greatest predictive value was one greater than or equal to 9 in the presence of a fasting glycemic index of 100 mg/dL 14.

Although various scales similar to FINDRISC exist, this one is perhaps the most used worldwide and the most appropriate for use in our population 15.

In Colombia, a modified version of FINDRISC has been validated, adjusting the cutoff points of waist circumference to the appropriate values for diagnosing abdominal obesity in the Latin American population (94 cm for men and 90 cm in women) 16. This measure exhibited a sensitivity of 74% and a specificity of 60% for detecting changes in glucose regulation (diabetes or pre-diabetes) with a score greater than or equal to 12 in a population of volunteers with no diagnosed diabetes 17.

These values suggest that the number of false negatives is low, and according to the information available in the literature, these patients can be expected to present a very low rate of complications during the following three years 18, at which point screening should again be carried out. False positives do not represent a significant impact on the emotional state (anxiety or depression) of patients 19.

Once the test has been conducted and a high FINDRISC score confirmed (cutoff point of 12), the fasting glycemic index will be measured as a first diagnostic test, and treatment will proceed according to the results of the diagnostic process. In cases where DMT2 is not confirmed, by the simple fact of the patient's having filled out the screening questionnaire, a first phase of education has effectively been performed with regard to good life habits, with beneficial results for the population as a whole. In the discussion about recommendations for the guide, it has been commented that if FINDRISC becomes a self-administered test, the replicability and validity of the muscular mass and abdominal circumference measurements will be less than when these are performed by a competent professional, and therefore it is preferable that the test be administered by the latter. 

The group developing the guide also performed an economic evaluation to determine the best screening strategy for Colombia 20. This evaluation compared schemes beginning with the fasting glucose test with schemes beginning with administration of the FINDRISC questionnaire. The screening strategy including FINDRISC plus the fasting glucose test in patients with a score greater than 12, supplemented by an OGTT for those patients with a glycemic score between 100 and 125 mg/dL, was the one that proved most cost-effective for Colombia and constitutes the recommendation for using FINDRISC in our environment.

**Recommendations. **

**Figure 1.**
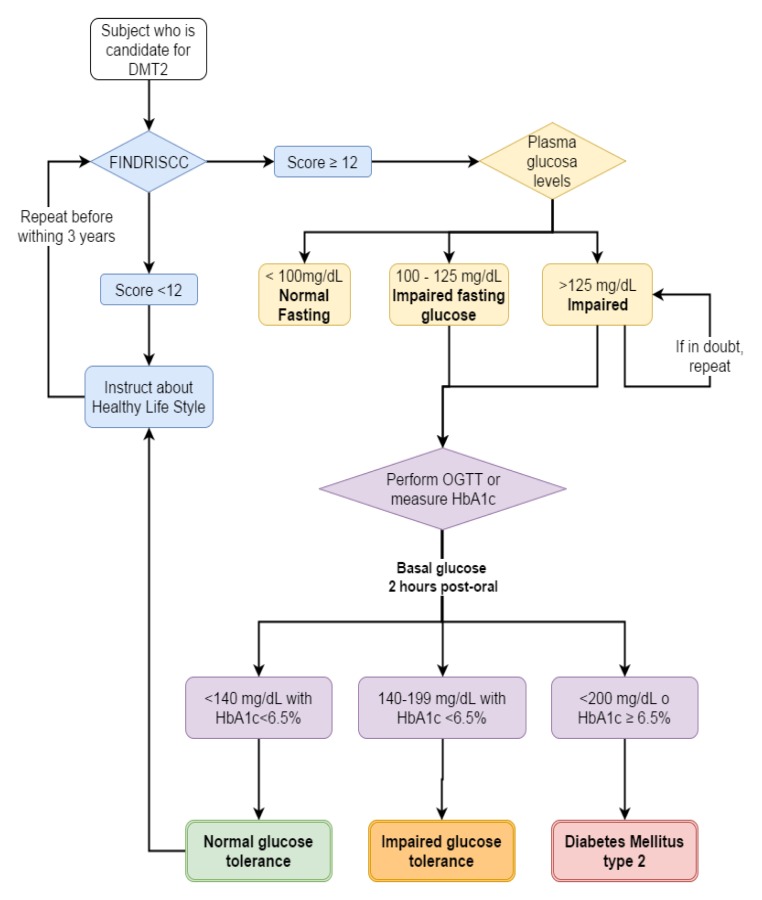
Algorithm to screening and diagnosis of diabetes mellitus type 2. DMT2: diabetes mellitus type 2. FINDRISCC: FINnish Diabetes Risk SCore, OGTT: oral glucose tolerance test, HbA1c: glycosylated hemoglobin

1. The use of FINDRISC with a cutoff point of 12 is recommended as a screening method for DMT2 among adults in Colombia (Fig. 1)**.**

**Recommendation strongly in favor.** Quality of evidence is moderate. ⴲⴲⴲⴱ

**Guidelines for good clinical practice**.

✔ FINDRISC must be used by people who are familiar with the tool. supplementary material shows the FINDRISC questionnaire adapted to the Colombian population. 

✔ Diagnostic testing for diabetes mellitus must be performed on all persons having a FINDRISC score ≥12.

✔ The most appropriate diagnostic test is measurement of the fasting plasma glycemic index.

✔ If the patient prefers, the fasting plasma glycemic index can be used as an initial screening and diagnostic test for diabetes mellitus.

✔ In those with a FINDRISC score ≥12 but who do not satisfy the diagnostic criteria for diabetes mellitus, it is recommended to define categories representing a higher risk of diabetes (prediabetes)* to include these people in DMT2 prevention programs.

* Categories at higher risk for diabetes:

- Abnormal fasting glycemic index: between 100 and 125 mg/dL.

- Glucose intolerance: glycemic index after 2 hours between 140 and 199 mg/dL in an OGTT.

✔ In all cases, treatment must include education on healthy lifestyles, emphasizing control of those DM risk factors that were identified in the questionnaire.

✔ Those with a FINDRISC score less than 12 should receive instruction on the importance of healthy lifestyles and undergo another screening in 3 years.

**Clinical question 2. ¿Should the HbA1c) be used in place of the oOGTT to diagnose diabetes?.**

**Answer to question 2. **A meta-analysis comparing the diagnostic capability of HbA1c with that of the OGTT as the test of reference 21 showed that HbA1c is an adequate tool for confirming the diagnosis of diabetes mellitus. In patients who score positive on a screening test, HbA1c with its high specificity (95.6%) can provide a reasonable basis for discarding the diagnosis in patients who do not have diabetes.

The most serious limitation of HbA1c is its only moderate sensitivity of 51.8%, meaning that a significant percentage of patients with diabetes mellitus will not be detected by this test. It is considered that if the patient shows a very high risk of diabetes in the screening test, an OGTT should be performed if the HbA1c is less than 6.5%.

Using the evidence described above, the GRADE methodology 22 for questions about diagnostic tests was applied to evaluate the potential impact of the test results on the final outcomes for patients who were erroneously classified as false positives (anxiety, depression) and false negatives (mortality and macro- and microvascular complications). As explained for clinical question 1, no significant impact of anxiety or depression was found in false positive patients. Based on results from the UKPDS 23 and ADDITION 24 studies, it was found that the 3-year impact of an inadequate diagnosis was not significant, with similar rates of death and micro- and macrovascular complications for misdiagnosed patients and for those who were diagnosed correctly and received pharmacological treatment. The 3-year impact was therefore taken into account, assuming that these patients would be followed up with further screening at this frequency at least.

In the economic evaluation to determine the most appropriate screening strategy in Colombia, as described under question 1, measurement of the glycemic index 2 hours after OGTT was compared with measurement of HbA1c as a DM diagnostic method for cases with high FINDRISC scores and abnormal fasting glycemic index. In this diagnostic sequence, the OGTT and not the HbA1c test was the most cost-effective strategy for Colombia 20. However, the HbA1c test is of fundamental importance in deciding on initial treatment and should be administered to all subjects with a DM diagnosis (as will be seen later). In this context, its cost-effectiveness may change if the probability of DM is very high.

**Recommendations **(Fig. 1).

2. It is recommended to use the HbA1c as a strategy for diagnosing diabetes mellitus in patients with a fasting plasma glycemic index between 100 and 125 mg/dL. It may also be used to corroborate the diagnosis when the result of the fasting plasma glycemic index is equivocal*. A value ≥6.5% confirms the diagnosis.

**Weak recommendation in favor of use** *. *Low quality of evidence ⴲⴲⴱⴱ

3. It is recommended to use the OGTT as a strategy for diagnosing diabetes mellitus in patients with a fasting plasma glycemic index between 100 and 124 mg/dL who express their preference for this strategy. It may also be used to corroborate the diagnosis when the result of the fasting plasma glycemic index test is equivocal*. A value ≥200 mg/dL 2 hours after ingesting a 75 g dose of glucose confirms the diagnosis.

**Weak recommendation in favor of use*. ***High quality of evidence. ⴲⴲⴲⴲ

* The fasting plasma glycemic index test is considered equivocal when the two measurements of glycemic index are divergent, that is, when one is greater than 125 and the other less.

**Good clinical practice guidelines.**

✔ Laboratories that perform the HbA1c test must comply with international standards, guaranteeing that the kits available in the country and the methods used are certified by the NGSP (National Glycohemoglobin Standardization Program - www.ngsp.org).

✔ In cases where clinical suspicion of diabetes mellitus is high in the screening test and the HbA1c is lower than 6.5%, an OGTT should be performed to confirm the diagnosis or to establish the presence of factors increasing the risk of diabetes (prediabetes)* so that such patients can be included in DMT2 prevention programs.

* Factors increasing the risk of diabetes:

- Abnormal fasting plasma glycemic index between 100 and 125 mg/dL

- Glucose intolerance: Glycemic index 2 hours after glucose administration of between 140 and 199 mg/dL in an OGTT.

✔ The OGTT test detects more cases of diabetes and consequently is more cost-effective. It should be used when the objective is to diagnose as many people as possible who have diabetes. 

✔ In all cases where diabetes mellitus is diagnosed, HbA1c should be measured initially so that the results can be used in decision-making about treatment and later to evaluate treatment effectiveness (see clinical question 4).

✔ In all cases where a diabetes mellitus diagnosis is rejected, education should take place regarding healthy lifestyles, emphasizing control of those risk factors for diabetes mellitus that were initially identified during screening.

### Topic 2. Initial treatment of diabetes type 2

**Assumptions. **The fundamental objective of treatment is to achieve fundamental lifestyle changes that lead to long-term metabolic control through weight normalization and maintenance and a steady increase in physical activity. The diet should be varied and balanced, taking into account the age and activity level of the subject. 

Consumption of foods that are sources of simple carbohydrates and saturated and trans fats should be reduced. These can be replaced by fats from fish and vegetable sources, preferably mainly monounsaturated fats such as canola and olive oils.

Consumption of whole fruits and vegetables should be increased because they provide fiber and antioxidants. Legumes are also sources of protein and fiber, and consumption should be encouraged, taking into account their higher calorie content.

Management of patients with DMT2 should be multifaceted to achieve adequate control of all risk factors, including hyperglycemia, dyslipidemia, high blood pressure, and tobacco abuse.

All patients with DMT2 should enter an educational program that supports them in changing their lifestyles, reaching their therapeutic objectives, and preventing complications of diabetes. This educational program must be ongoing and be led by a health professional certified in diabetes education, with the support of other healthcare professionals in fields such as nutrition, nursing, physical education, psychology, podiatry, and odontology.

Pharmacological treatment usually begins with oral antidiabetic medications when the patient is clinically stable, even if he/she has a very high HbA1c value. The decrease in HbA1c is directly proportional to the extent to which it initially was elevated. 

When the patient is highly symptomatic and clinically unstable, with severe weight loss, signs of dehydration, evidence of ketosis, and very high glycemic index, it is recommended to start insulin initially, although it can then be withdrawn gradually.

Patients with DMT2 and excess weight should enter a program that will help them to follow a diet with the necessary reduction in caloric intake for them to lose weight and reach a body mass index of approximately 25 kg/m^2^.

The concomitant initial use of weight loss medications, including some Glucagon-like peptide-1 (GLP-1) receptor agonists that also have an antihyperglycemic effect, may be indicated in patients with DT2 and obesity (BMI ≥30 kg/m²). 

When a patient with DMT2 is morbidly obese, with a BMI ≥35 kg/m², it can be beneficial to enroll the patient in a program directed towards bariatric surgery, provided that other criteria are in favor, there are no contraindications, and the patient shows a willingness to make fundamental lifestyle changes.

**Clinical question 3. In adults recently diagnosed with DMT2, can disease management begin only with fundamental lifestyle changes?.**

**Answer to question 3.** The UKPDS has been the largest study with the most intensive follow-up of persons with DMT2 and was designed to establish the effectiveness of intensive initial glycemic control over diabetic complications 5. Intensive management began with sulfonylurea or basal insulin and conventional management with diet alone. Although glycemic control deteriorated in both groups, a difference in HbA1c of 0.9% was maintained, with the intensive treatment group maintaining an average HbA1c of 7%. This amounted to a significant reduction in the incidence of diabetic complications and in particular of macrovascular complications. In a subgroup of overweight patients, intensive treatment began with metformin, and although this group maintained a slightly higher average HbA1c (7.4%) and the difference from the control group was only 0.6%, this group experienced the greatest reduction in incidence of all diabetes-related complications and a signification reduction in mortality. Weight in this group of patients remained very similar to that of the control group 24. Based on these results, clinical practice guidelines now propose that intensive DMT2 treatment begin with lifestyle changes and preferably with metformin. 

A recent study of high methodological quality determined the impact of a multifactorial intervention with intensive dietary changes and exercise along with the usual treatment and diabetes education, with a focus on losing weight, in patients with DMT2 of moderate duration (approximately 5 years) 25. Although participants succeeded in losing 8.6% of their body weight at the end of the first year and 6% at the end of the study (vs. 0.7% and 3.5% in the control group) and achieved small changes in HbA1c, there was no significant reduction in the incidence of fatal or nonfatal cardiovascular events, and the study was prematurely terminated because the results indicated the approach was futile. It is important to note that the initial benefits of fundamental lifestyle changes (weight loss, decrease in HbA1c) began to be lost after the first year.

When the impact of each lifestyle change is evaluated on an individual basis, the evidence shows that physical activity has a favorable impact on the risk of death. A cohort of subjects with DMT2 followed for an average of 9.4 years 26 demonstrated a significant tendency to reduce their risk of total and cardiovascular mortality with higher levels of total physical activity, as measured by a scale, and with higher intensity of physical activity during their leisure time, as measured in mets-h/week. However, more time spent walking did not reduce risk. The lowest risk of total (HR= 0.62, CI 95%= 0.49-0.78) and cardiovascular (HR= 0.51; CI 95%= 0.32-0.81) mortality was observed in moderately active individuals.

In a meta-analysis of five studies including the preceding one, the risk of death by all causes was less for those with high total physical activity levels (HR= 0.60; CI 95%= 0.49-0.73). Structured exercise programs have a major impact on glycemic control compared to simple recommendations to perform physical exercise (reduction in HbA1c= -0.73%; CI 95%= -1.06%-0.40% vs. -0.57%; CI 95%= -1.14%-0.01% respectively) 27. The effect was seen both with aerobic exercise and resistance training, and the combination of both types of exercise produced a similar effect. In addition, this effect became greater as the number of sessions per week increased, showing an additional reduction in HbA1c of -0.39% for each weekly session added.

As for evaluations of structured nutritional interventions, a recent meta-analysis of 16 studies with follow-up periods from 6 months to 4 years showed that diets low in carbohydrates, with low glycemic indices, and with high protein content or of the Mediterranean type, all improved glycemic control significantly over the comparison group. However, the Mediterranean diet produced the largest decrease in HbA1c (-0.47% on average, *p=* <0.00001) and the largest weight loss (-1.84 kg on average, *p=* <0.00001) 28. In addition, a high-quality controlled and randomized clinical study demonstrated that the Mediterranean diet significantly reduced the risk of major cardiovascular events (acute myocardial infarction, stroke, or death from cardiovascular causes) (HR= 0.70; CI 95%= 0.54-0.92) in subjects with high cardiovascular risk followed for an average of 4.8 years. Almost half the study group had diabetes, but no interaction was demonstrated with the presence or absence of this condition in the subgroup analysis 29.

With this information in hand, the group developing the guide considered that the evidence in favor of initiating treatment with fundamental lifestyle changes as the only initial management strategy in patients with recently diagnosed DMT2 was insufficient. In addition, it was realized that good-quality information exists indicating that initiating pharmacological treatment with metformin in patients with recently diagnosed DMT2 helps to reduce cardiovascular incidents over the long term.

Therefore, it was concluded that the available evidence was sufficient to demonstrate clinical benefits for patients in combining pharmacological treatment with therapeutic lifestyle changes from the beginning. 

**Figure 2:**
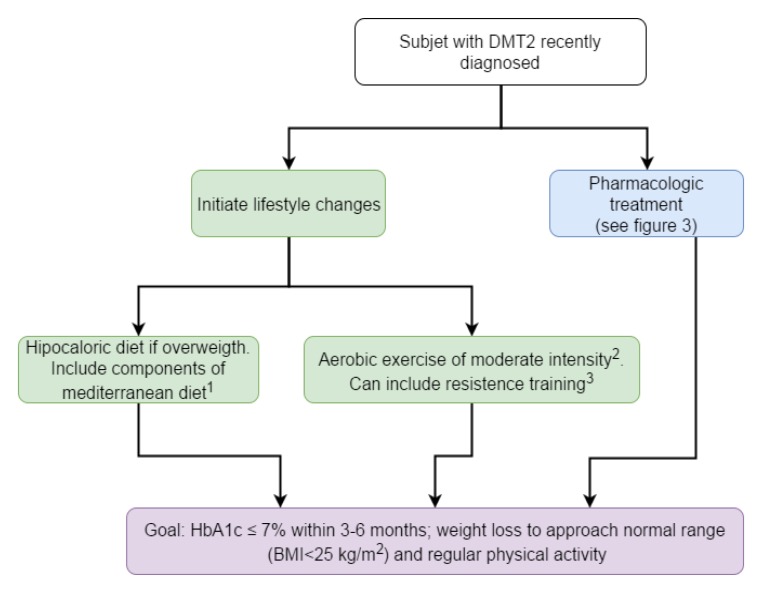
Algorithm to initial management of patients with diabetes mellitus type 2. ^1^ The mediterranean diet adapted to our surroundings must include mainly vegetables, legumes, whole-grain foods, fresh and dry fruits, olive oil, nuts; also moderate consumption of fish, poultry and low fat dairy products. Consumption of red meat, eggs, milled grains and sugars must be sporadic. ^2^ Aerobic exercise includes activities such as riding a bicycle, walking, swimming, dancing and repeated rythmic movements (≥ 10) of each muscular group. The frequency must be ≥ 150 minutes per week, and the intensity should not exceed a heart rate equal to (220 − age) × 0,7. ^3^ Resistance training include weight lifting routines which should be gradual in the amount of weight and the frequency. HbA1c: glycosylated hemoglobin, BMI: Body Mass Index

**Recommendations** (Fig. 2)

4. In patients with recently diagnosed DMT2, initial treatment with lifestyle changes only is not recommended. 

**Strong recommendation against. **Quality of evidence moderate**. **ⴲⴲⴲⴱ

5. In patients with recently diagnosed DMT2, it is recommended to begin pharmacological treatment with metformin simultaneously with lifestyle changes, although the initial HbA1c value is of value in achieving the treatment objectives.

**Strong recommendation in favor.**Quality of evidence moderate. ⴲⴲⴲⴱ

6. In patients with recently diagnosed DMT2, it is suggested that lifestyle changes include the components of a Mediterranean diet. 

**Weak recommendation in favor. **Quality of evidence moderate. ⴲⴲⴲⴱ

7. In patients with recently diagnosed DMT2, it is suggested that lifestyle changes include moderate-intensity aerobic exercise.

**Weak recommendation in favor. **Quality of evidence low. ⴲⴲⴲⴱ

8. In patients with recently diagnosed DMT2, it is suggested that lifestyle changes include resistance training in cases where the patient prefers it.

**Weak recommendation in favor. **Quality of evidence very low**. **ⴲⴲⴱⴱ

**Good clinical practice guidelines**.

When lifestyle changes are initiated, the following factors should be taken into account: 

✔ It is recommended to adapt the Mediterranean diet to the characteristics of the patient's environment, conserving the predominance of consuming vegetables, garden produce, whole-grain cereals, whole fruits, dried fruits, olive oil, and nuts. The diet includes moderate consumption of fish, poultry, low-fat dairy products, and wine with meals if this is habitual for the patient. Consumption of red meat, eggs, and refined grains should be sporadic.

✔ Habitual consumption of alcohol should not be encouraged.

✔ A structured exercise program should be established to achieve a favorable impact.

✔ Aerobic exercise includes activities such as riding a bicycle, walking, swimming, dancing, and repeated rhythmic movements (≥10) of the same muscle group. The frequency should be equal to or greater than 150 minutes per week, and the intensity must not exceed a cardiac rate equal to (200 - age) × 0.7. To achieve a weight-loss effect, exercise must be much more frequent.

✔ Resistance training should include weight routines that increase gradually the amount of weight and the frequency.

✔ For patients with recently diagnosed DMT2 who have physical limitations that impair mobility, it is suggested to individualize the exercise prescription and to have it validated by a physicist or a sports medicine specialist.

✔ If the patient is clinically unstable, it is preferable to postpone the beginning of the exercise program until symptoms are clinically controlled.

✔ Metformin should be administered gradually, beginning with 500 mg per day and increasing to 1,000 mg twice a day to avoid gastrointestinal intolerance (nausea, abdominal pain, diarrhea). If gastrointestinal intolerance occurs, it can be minimized by taking the medication with meals and using the prolonged-release format. 

✔ The dose of metformin must be reduced to a maximum of 1,000 mg per day if the glomerular filtration rate falls below 50 mL/min and must be discontinued if it falls below 30 mL/min. The same contraindication exists when there is a severe risk of lactic acidosis, for example in states of severe hypoxemia, liver failure, or alcoholism. 

✔ When metformin is contraindicated or not tolerated, it can be replaced as an initial treatment by any of the other oral antidiabetic medications approved for monotherapy.

**Clinical question 4. ¿In adults with recently diagnosed DMT2, when should treatment with more than one medication be undertaken to achieve adequate glycemic control?.**

**Answer to question 4. **Upon evaluation of the available evidence on combinations of medications as an initial pharmacological treatment in patients with DMT2, all the medications evaluated achieve significant HbA1c reductions of similar magnitude when combined with metformin as an initial treatment (reduction in HbA1c= -0.43%; CI 95%= -0.56-0.30) 30. Along the same lines, all the oral antidiabetic medications significantly increase the probability of achieving glycemic control goals (Relative risk to reach HbA1c= <7%: 1.40; CI 95%= 1.33-1.48) 30.

It should be emphasized that none of the combined therapies is supported by evidence suggesting an impact on the risk of microvascular complications, and evidence of the impact on cardiovascular risk is weak. 

As a consequence of this information, the group developing the guide considers that there is sufficient evidence in favor of initiating pharmacological treatment with combined therapy in patients with recently diagnosed DMT2 to obtain better metabolic control. This approach is suggested in cases in which at the time of diagnosis, the HbA1c value is greater than 8%. This was the minimum average value for the patients included in these studies and is more than one percentage point above the goal of 7%, at which point the treatment can be reduced to metformin as monotherapy.

Given the similarity in the effectiveness of the various medications, it is considered of fundamental importance to evaluate the risks of adverse effects when defining the medication to combine with metformin. The risk of hypoglycemia has been found to increase significantly in initial combinations with metformin as opposed to metformin alone (RR= 1.56; CI 95%= 1.08-2.26). However, this increment disappears in the sensitivity analysis when studies using sulfonylureas or glinides are excluded and only combinations of metformin with thiazolidinediones, dipeptidyl peptidase 4 (DPP4) inhibitors, and Sodium glucose co-transporter 2 (SGLT2) inhibitors are taken into account (RR= 1.20; CI 95%= 0.91-1.56) 30.

Combined therapy using metformin with sulfonylureas is associated with a statistically and clinically significant increase in the risk of hypoglycemia compared with monotherapy with metformin (34). This risk is greater when the combination includes glibenclamide (RR= 16.05; CI 95%= 6.22-41.39), glimepiride (RR= 2.08; CI 95%= 0.74-5.86), or glicazide (RR= 4.09; CI 95%= 2.13-7.89) 31.

As for body weight changes, which are another effect that can be adverse or beneficial during initial treatment, controlled clinical studies have demonstrated that the combination of metformin and thiazolidinedione increases weight 32, whereas that of metformin with SGLT2 inhibitors reduces it 33. In a meta-analysis of the initial combination of metformin with DPP4 inhibitors, it was observed that this combination reduces the magnitude of body weight loss compared to metformin alone (difference= 0.44, CI 95%= 0.22-0.67), but the absolute effect was still a loss in weight 34. 

Other potential adverse effects were also taken into account such as the risk of cardiac failure, factures, and bladder cancer associated with thiazolidinediones 35-37 and that of urogenital tract infections associated with SGLT2 inhibitors 38.

The evidence presented suggests that the best risk-benefit profile is that of combined therapy with metformin and a DPP4 inhibitor. Other reasonable options are the combination of metformin with a sulfonylurea having low risk of hypoglycemia (glimepiride or glicazide) or the combination of metformin and a SGLT2 inhibitor.

**Figure 3.**
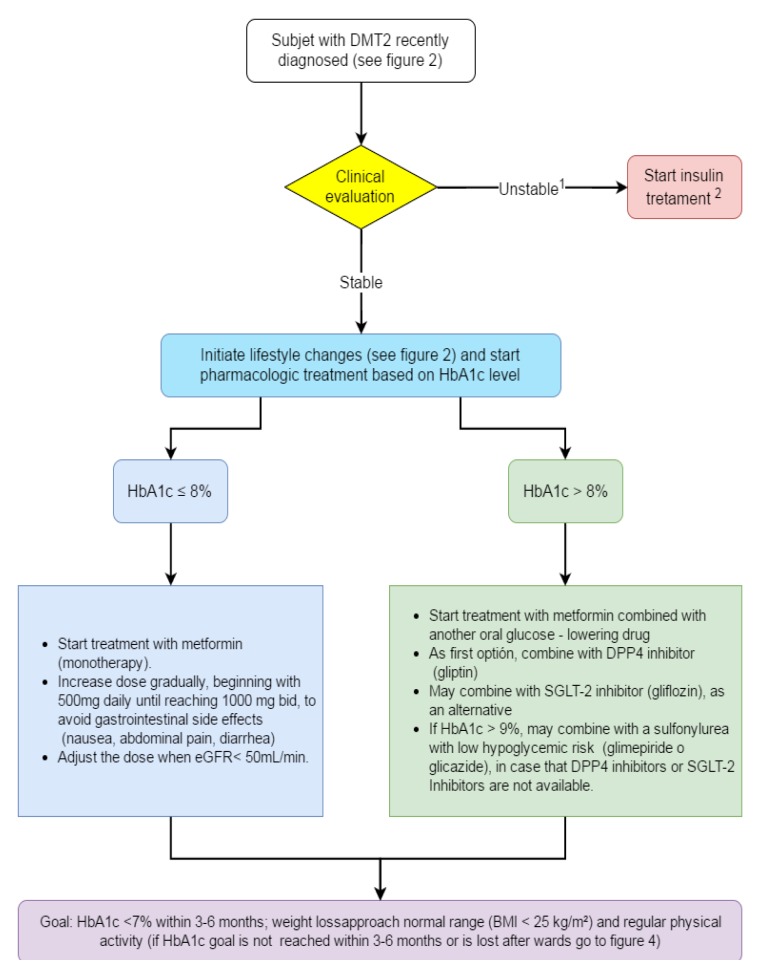
Algorithm to initial pharmacologic treatment. ^1^ Clinically unstable: very symptomatic, with acute weight loss, signs of dehydration, evidence of ketosis and very high blood glucose. ^2^ May require insulin in combination with other glucose- lowering drugs or in intensified regimes. Insulin use may be transient. HbA1c: glycosylated hemoglobin, GFR: glomerular filtration rate DPP4: dipeptidyl peptidase 4, SGLT-2: Sodium glucose co-transporter 2, BMI: Body Mass Index.

**Recommendations** (Fig. 3).

9. In patients with recently diagnosed DMT2 and HbA1c levels >8%, it is recommended to administer a combined therapy from the beginning with metformin and another oral antidiabetic medication.

**Strong recommendation in favor. **Quality of evidence moderate. ⴲⴲⴲⴱ

10. In patients with recently diagnosed DMT2 for whom it is decided to administer a combined treatment from the beginning, the combination of metformin and a DPP4 inhibitor is recommended.

**Strong recommendation in favor. **Quality of evidence moderate. ⴲⴲⴲⴱ

11. In patients with recently diagnosed DMT2 for whom it is decided to administer a combined treatment from the beginning, the combination of metformin and a SGLT2 inhibitor is suggested as an alternative to the combination of metformin and a DPP4 inhibitor.

**Weak recommendation in favor. **Quality of evidence moderate. ⴲⴲⴲⴱ

12. In patients with recently diagnosed DMT2 and HbA1c >9% who cannot use the combination of metformin with DPP4 or SGLT2 inhibitors, the combination of metformin with a sulfonylurea having low risk of hypoglycemia (glimepiride or glicazide) is recommended. 

**Weak recommendation in favor. **Quality of evidence moderate. ⴲⴲⴲⴱ

13. In patients with recently diagnosed DMT2, it is suggested *not to use* the combination of metformin and glibenclamide because of the high risk of hypoglycemia. 

**Weak recommendation against. **Quality of evidence moderate. ⴲⴲⴲⴱ

14. In patients with recently diagnosed DMT2, it is suggest *not to use* the combination of metformin and thiazolidinedione because of the increased risk of edema, cardiac failure, and fractures.

**Strong recommendation against. **Quality of evidence moderate**. **ⴲⴲⴲⴱ

**Good clinical practice guidelines**.

✔ Fixed combinations of metformin with another oral antidiabetic medication should be preferred whenever available because they improve compliance. 

✔ The dosage of DPP4 inhibitors should be adjusted if the glomerular filtration rate falls below 50 mL/min, with the exception of linagliptine. 

✔ For patients with DMT2 in whom sulfonylureas are used, it is recommended to emphasize education and implement glucose self-monitoring to detect and treat appropriately any episodes of hypoglycemia. 

✔ Use of sulfonylureas is not recommended if the glomerular filtration rate drops below 30 mL/min, except for glipizide.

✔ It is recommended to watch for and treat genitourinary tract infections promptly when SGLT2 inhibitors are used.

✔ The effectiveness of SGLT2 inhibitors tends to decrease as renal function deteriorates, and the package insert should be consulted to determine the opportune moment to discontinue them. 

✔ If episodes of hypoglycemia occur when sulfonylureas are used, a change to a medication regime that does not cause hypoglycemia should be considered.

### Topic 3: failure of initial treatment

**Assumptions. **The fundamental objective of pharmacological therapy is to achieve adequate metabolic control, maintaining HbA1c at the desired level without causing adverse effects and without negative interference with lifestyle changes.

If efforts to reduce HbA1c to the goal value do not succeed in 3 to 6 months with the initial treatment, or if after the goal is reached, HbA1c rises again, treatment must be intensified by adding another antidiabetic medication.

**Clinical question 5. ¿In patients with DMT2 who have already started pharmacological treatment with metformin and have not reached their control goals, which of the following antidiabetic medications should be added to the treatment: sulfonylureas, DPP4 inhibitors, thiazolidinediones, GPL1 analogs, SGLT2 inhibitors, or basal insulin?.**

**Answer to question 5. **Upon reviewing the evidence with respect to second-line therapy in patients with DMT2 that is not controlled by high doses of metformin, it is apparent that various therapeutic options (sulfonylureas, glinides, thiazolidinediones, DPP4 inhibitors, SCLT2 inhibitors, basal insulin, and biphasic insulin) are effective in combination with metformin 39-41. In comparison with addition of a placebo, HbA1c decreased by between 0.6% and 1%, and the probability of achieving the goal of 7% or less was 2 to 3 times higher.

The differences among these combinations are rooted mainly in their profiles of adverse events and their safety in terms of hypoglycemia and cardiovascular pathology among others. The incidence of hypoglycemia, which is an important component of safety, increased significantly with addition of sulfonylureas, glinides, or insulin, but did not increase significantly with alfa-glucosidase, DPP4 or SGLT2 inhibitors, GLP1 agonists, or thiazolidinediones 39-41. Among the various sulfonylureas, glibenclamide produced 4 to 8 times more hypoglycemia than glimepiride or gliclazide 31. The risk of severe hypoglycemia also increased more than threefold with sulfonylureas, especially when HbA1c was lower and BMI was higher 42.

As for effect on body weight, addition of sulfonylureas, glinides, thiazolidinediones, or insulin led to increases between 1 and 3 kg, with the greatest increases occurring with thiazolidinediones and biphasic insulin 39-41. DPP4 inhibitors generally had no effect on weight. SGLT2 inhibitors can produce a weight loss of 2 to 3 kg 39. The largest weight reduction occurred with GLP1 receptor agonists and ranged from 1.4 to 3.7 kg 39-44. 

In generating the recommendation, the evidence already mentioned in the context of the previous question was taken into consideration, suggesting an increased risk of cardiac failure, fractures, and bladder cancer associated with use of pioglitazone. 

Recent long-term studies have been published of the cardiovascular safety of new classes of medications. None of the three studies involving DPP4 inhibitors showed an increased risk of cardiovascular events (death due to cardiovascular causes, nonfatal infarcts, nonfatal strokes, hospitalization for unstable angina) in a wide spectrum of patients with DMT2 (primary prevention with high-risk patients, secondary prevention, recent acute coronary syndrome) 45-47. Only one study (with saxagliptine) showed an increase in hospitalizations for cardiac failure 47. There was no significant increase in the incidence of pancreatitis or pancreatic cancer, although the numbers were very low.

Another study with a SGLT2 inhibitor also demonstrated no increase in risk for myocardial infarctions or for cerebrovascular events. On the contrary, early and highly significant reductions were observed for death due to cardiovascular causes (HR= 0.62; CI 95%= 0.49-0.77), death from all causes (HR= 0.68; CI 95%= 0.57-0.82), and hospitalization for cardiac failure (HR= 0.65; CI 95%= 0.50-0.85) 48. 

An economic cost-effectiveness analysis has upheld these conclusions 49. The combinations of metformin with sulfonylureas, DPP4 inhibitors, and pioglitazone are cost-effective, whereas that with a GLP-1 receptor agonist (exenatide) was not. When this analysis was done, other GLP-1 receptor agonists were not included, nor were SGLT2 inhibitors, because safety information for these medications was not yet available. However, preliminary results on cardiovascular safety could justify new cost-effectiveness evaluations.

Taking into consideration the pharmacological options available in the Colombian environment, the first choice for second-line therapy is considered to be the combination of metformin with a DPP-4 inhibitor because this group of medications had the best effectiveness and safety profile.

As a second option, treatment with SGLT-2 inhibitors could be considered, although presently available studies do not provide sufficient information to prefer this therapy over DPP-4 inhibitors. At the time of this guide, no SGLT-2 inhibitor had been approved by INVIMA (Colombia National Food and Drug Surveillance Institute). 

**Figure 4.**
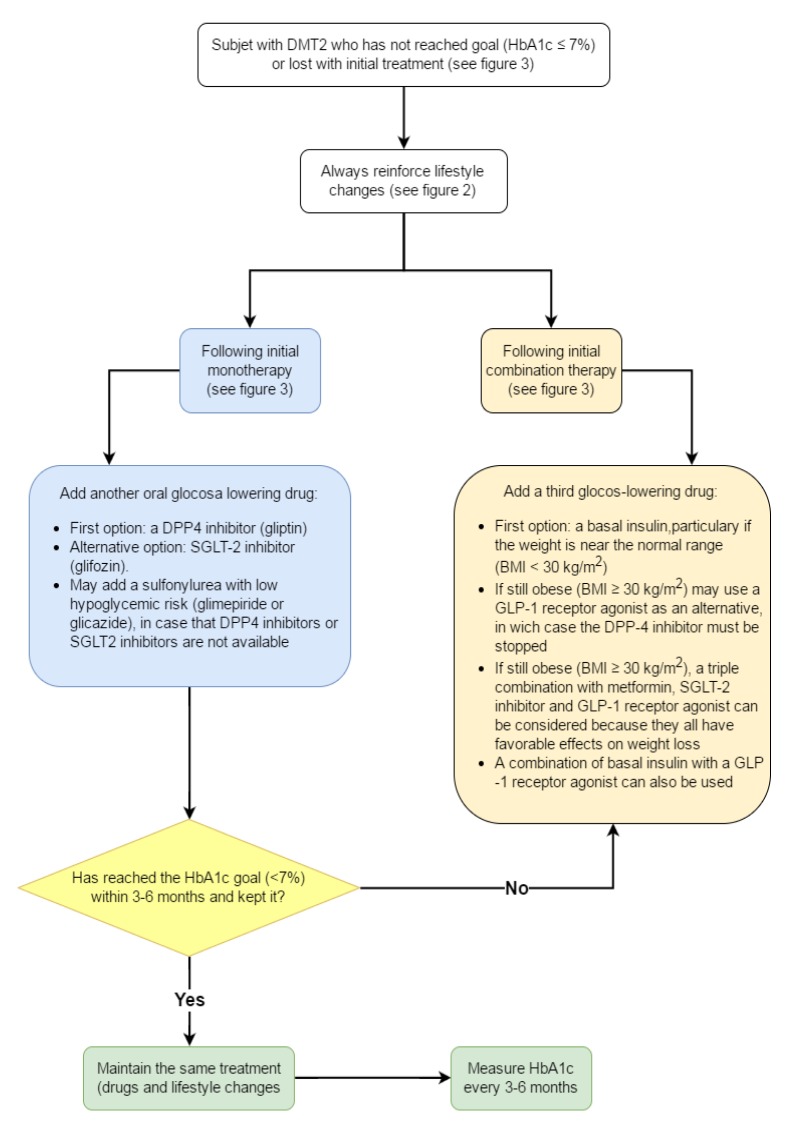
Pharmacologic treatment when goal is not reached or is lost with initial treatment. DPP4: dipeptidyl peptidase 4, SGLT-2: Sodium glucose co-transporter 2, BMI: Body Mass Index, GLP-1: Glucagon-like peptide-1, HbA1c: glycosylated hemoglobin

**Recommendations** (Fig. 4).

15. In patients with DMT2 who did not reach their therapeutic goal or failed to maintain it with metformin as a monotherapy (Hb1Ac <7%), addition of a second oral antidiabetic medication is recommended. 

**Strong recommendation in favor. **Quality of evidence moderate. ⴲ ⴲ ⴲ ⴱ

16. In patients with DMT2 who did not reach their therapeutic goal or failed to maintain it with metformin as a monotherapy (Hb1Ac <7%), it is recommended as a first step to add a DPP-4 inhibitor. 

**Strong recommendation in favor. **Quality of evidence moderate. ⴲ ⴲ ⴲ ⴱ

17. In patients with DMT2 who did not reach their therapeutic goal or failed to maintain it with metformin as a monotherapy (Hb1Ac <7%), it is suggested to add a SGLT2 inhibitor as an alternative to a DPP-4 inhibitor. 

**Weak recommendation in favor. **Quality of evidence moderate. ⴲ ⴲ ⴲ ⴱ

18. In patients with DMT2 who did not reach their therapeutic goal or failed to maintain it with metformin as a monotherapy (Hb1Ac <7%), it is suggested to add a sulfonylurea having low risk of hypoglycemia (glimepiride, glicazide) when DPP4 or SGLT2 inhibitors are not available or are contraindicated. 

**Weak recommendation in favor. **Quality of evidence moderate. ⴲ ⴲ ⴲ ⴱ

19. It is suggested not to add glibenclamide to the treatment of patients with DMT2 who have not reached their therapeutic goal or have failed to maintain it with metformin as a monotherapy, because of the high risk of hypoglycemia.

**Weak recommendation against. **Quality of evidence moderate. ⴲ ⴲ ⴲ ⴱ

20. It is suggested not to add thiazolidinediones to the treatment of patients with DMT2 who have not reached their therapeutic goal or have failed to maintain it with metformin as a monotherapy, because of the higher risks of edema, cardiac failure, and fractures.

**Weak recommendation against. **Quality of evidence moderate. ⴲ ⴲ ⴲ ⴱ

21. In patients with DMT2 who did not reach their therapeutic goal or failed to maintain it with metformin as a monotherapy (Hb1Ac <7%) and who remain obese (BMI= ≥30 kg/m²), addition of a GLP-1 agonist is recommended because of its beneficial effect on weight loss.

**Strong recommendation in favor. **Quality of evidence moderate. ⴲ ⴲ ⴲ ⴱ

**Good clinical practice guidelines.**

✔ Every time that pharmaceutical treatment fails to reach or maintain therapeutic goals, lifestyle changes must be reviewed and intensified.

✔ Intensified lifestyle changes must include significant weight loss. In this respect, it should be remembered that sulfonylureas and thiazolidinediones increase weight, DPP4 inhibitors do not affect it, metformin can reduce weight, and both SGLT2 inhibitors and GLP1 receptor agonists reduce it.

✔ Education should be offered from the beginning to patients requiring sulfonylureas to prevent, detect, and treat promptly any episodes of hypoglycemia.

✔ If hypoglycemic events occur when sulfonylureas are added, a change to medications that do not cause hypoglycemia should be considered.

✔ Addition of a GLP-1 receptor agonist requires educating the patient in the use of subcutaneous injection and calibration of the dose, which must be increased gradually to minimize gastrointestinal effects such as nausea and vomiting.

✔ If the patient is highly symptomatic and clinically unstable, with acute weight loss, signs of dehydration, evidence of ketosis, and a very high glycemic index, adding insulin is recommended. 

**Clinical question 6. ¿In patients with DMT2 who have already started pharmacological treatment with metformin and a second antidiabetic medication and have not reached their control goals, what antidiabetic medication is indicated as an addition to the treatment (as a third antidiabetic medication)?.**

**Answer to question 6. **A systematic review 50 has evaluated the effect of adding a third medication to the combination of glibenclamide and metformin on HbA1c levels, body weight, and occurrence of hypoglycemia. 

The addition of thiazolidinediones to treatment with glibenclamide and metformin was the combination that most effectively reduced HbA1c (-1.15%; CI 95%= -1.35-0.95%) compared to a placebo, followed by the GLP-1 receptor agonists (-1.04%; CI 95%= -1.24-0.85%), the DPP-4 inhibitors (-0.89%; CI 95%= -1.11-0.67%), and insulin (-0.77%; CI 95%= -0.95-0.47%), with all these results being statistically significant.

In comparison with insulin along with metformin and glibenclamide, the thiazolidinediones produced a more pronounced reduction in HbA1c which was statistically but not clinically significant (0.22%; CI 95%= 0.07-0.31%). The GLP-1 receptor agonists in comparison with insulin did not show a significant difference.

As for changes in body weight, GLP-1 agonists tend to reduce it and thiazolidinediones to increase it, but these changes are not statistically significant. The combination with insulin did increase weight to a statistically and clinically significant extent (2.31 kg; CI 95%= 0.13-4.48 kg). As for DPP-4 inhibitors, results from only one study were included, and this study did not consider body weight.

A network meta-analysis has also been performed comparing various medications as a third antidiabetic option 42. This analysis found that none of the medications analyzed (GLP-1 agonists, thiazolidinediones, DPP-4 inhibitors, and insulin) generated a statistically significant reduction of HbA1c levels when compared with one another. As for body weight, only the GLP-1 receptor agonists reduced it significantly (-1.63 Kg; CI 95%= -2.71-0.60), whereas the other medications showed no significant effect.

Information on severe hypoglycemic episodes was very limited and could not generate statistically significant results.

Another meta-analysis 51 was performed comparing critical outcomes like mortality, microvascular complications (nephropathy, retinopathy, neuropathy), macrovascular complications (stroke, myocardial infarction, peripheral arterial disease), abandonment of or adherence to treatment, and quality of life (noted as an important outcome). However, the data were not consistent in the various clinical studies, and the follow-up periods were relatively short, making it impossible to perform an analysis and draw conclusions. Still another meta-analysis evaluated the risk of major cardiovascular events with GLP-1 receptor agonists and with other antidiabetic medications 52 and found no statistically significant differences.

**Recommendations** (Fig. 4).

22. It is suggested to add basal insulin as a third antidiabetic medication if the combination of two pharmacological treatments does not enable the patient to reach and maintain the HbA1c goal and if the patient is not obese (BMI= <30 kg/m²).

**Weak recommendation in favor. **Quality of evidence moderate. ⴲ ⴲ ⴲ ⴱ

23. It is suggested to add a GLP-1 agonist as a third antidiabetic medication for patients who have failed to reach or maintain their HbA1c goals with oral combination therapy and who remain obese (BMI= ≥30 kg/m²). 

**Weak recommendation in favor. **Quality of evidence moderate. ⴲ ⴲ ⴲ ⴱ

24. The combination of metformin, a SGLT-2 inhibitor, and a GLP-1 agonist is recommended only for patients who have failed to reach or maintain their HbA1c goal with dual therapy and who remain obese (BMI= ≥30 kg/m²).

**Weak recommendation in favor. **Expert consensus.

**Good clinical practice guidelines**.

✔NPH insulin can be used as basal insulin if injected before going to bed, at 10 or 11 PM. 

✔Analogous prolonged-action insulins (glargina, detemir, degludec) carry less risk of hypoglycemia than NPH insulin and are therefore preferable if the objective is to maintain the patient at optimal control avoiding hypoglycemic episodes, or if such episodes occur and the intention is to reach glycemic control goals. These insulins are injected once a day, always at the same time, except for detemir, which can be administered twice a day if necessary.

✔The initial dose of basal insulin is 10 units per day or 0.2 units/kg body weight/day. Various algorithms exist to calibrate the dose, with all of them based on incrementing the dose 2 to 4 units at a time if the fasting glycemic index is above the preferred value on 2 or 3 successive days or the average over this same interval is high.

✔GLP-1 receptor agonists require a progressive dose increase to minimize gastrointestinal side effects, with each one having its own instructions to this effect. Each one also has its own form of administration, which may be once or twice a day or even weekly.

✔When the anticipated response is not obtained with basal insulin or GLP-1 receptor agonists, these two medications may be combined, or schemes of intensive insulin therapy can be used. However, in such cases, it is recommended that a specialized team evaluate the goals and the necessity of intensifying treatment for each patient.

✔During therapy with insulin in combination with oral antidiabetic medications that can cause hypoglycemia, the patient must be educated about early detection of hypoglycemic symptoms and in the corrective actions to be taken if it occurs. Special care must be taken in this matter with combinations involving insulin and sulfonylureas. If hypoglycemic episodes are occurring and insulin continues to be necessary, sulfonylurea should be replaced by another medication that does not cause hypoglycemia. 

### Topic 4. Goals of glycemic control

**Assumptions. **Reaching and maintaining an average HbA1c of 7% reduces the incidence of micro- and macrovascular complications of DMT2.

The normal analytic upper limit for HbA1c is around 6%, but controlled clinical studies have not shown that more intensive treatment to reach a normal level (<6.5%) produces benefits that justify the risks in most patients with DMT2.

Metabolic control in patients with DMT2 should be directed towards reaching and maintaining an HbA1c goal ≤7% without producing risks that outweigh the benefits and avoiding as much as possible any adverse effects and deterioration in quality of life.

In patients with DMT2, with no serious comorbidity factors, and aged 65 years or over, HbA1c levels can be lowered to 6.5%, especially if there is evidence of microangiopathy. As before, this should be done without producing risks that outweigh the benefits and avoiding as much as possible any adverse effects and deterioration in quality of life.

**Clinical question 7. ¿Do adults aged 65 years or over with DMT2 require an individualized glycemic control goal that differs from the goal of reaching a glycosyladic hemoglobin index equal to or less than 7%?.**

**Answer to question 7. **The evidence evaluated indicates that:

•Intensive treatment focused on achieving an HbA1c value close to normal does not impact the overall mortality risk or the risk of cardiovascular mortality, either in the general population or in the subgroup of those aged 60 years and over. However, there is heterogeneity in this latter group (RR= 1.13; CI 95%= 0.81-1.57, I²= 72%) 53.

•In a study that evaluated the benefits of intensive glycemic control (HbA1c <6%) for reducing cardiovascular risk, which had to be suspended because of an increase in mortality in the group with intensive control, subgroup analysis demonstrated that this increase was significant in those under 65 years of age, but not in those who were older (HR= 0.97; CI 95%= 0.7-1.36 for cardiovascular mortality and HR= 1.06; CI 95%= 0.84-1.34 for death from all causes).

•On the contrary, in this same study, a reduced incidence of nonfatal myocardial infarction was observed only in the subgroup younger than 65 years of age. The incidence of hypoglycemia requiring medical assistance was almost double in the group aged 65 years and over (annual incidence 4.45% vs. 2.45%), and in fact hypoglycemia occurred so often at the beginning of the study in participants over 80 years of age that inclusion of patients in this age group was suspended 54.

•Treatment focused on attaining an HbA1c value close to normal did not impact the risk of stroke in the general population of patients with DMT2. Similar findings resulted from a subanalysis considering only those over 65 years of age (RR= 1.01; CI 95%= 0.91-1.11) 55.

•No specific data are available on quality of life for those older than 65 years of age. However, data obtained from the general population suggest that intensive treatment has no impact on measures of their general state of health, symptoms associated with diabetes, or depression. 

•A recent large-scale review study (34,912 patients with DMT2 and ages ranging up to 72 years) did not succeed in performing the planned analysis for the group ≥65 years of age, but it found that in general, intensive glycemic control significantly reduces the incidence of nonfatal myocardial infarction (RR= 0.87; CI 95%= 0.77-0.98) and amputations (RR= 0.45; CI 95%= 0.45-0.94) based on moderate-quality evidence, but increased the risk of hypoglycemia (RR= 2.18; CI 95%= 1.53-3.11) based on high-quality evidence. Intensive control does not seem to affect the incidences of mortality, nonfatal cerebrovascular events, or terminal renal illness 56.

•Taking this information into consideration, the group developing the guide concluded that there is no significant benefit in administering intensive treatment focused on obtaining HbA1c values close to normal in patients older than 65 years of age, but that on the contrary, this group has a higher risk of hypoglycemia. For these reasons, the group decided to reject this approach.

Similarly, the group developing the guide discussed the fact that there is no evidence demonstrating that pursuing a more aggressive treatment goal improves the risk-benefit equation. Therefore, in patients older than 65 years of age without other comorbidity factors and who are functionally independent, the treatment goal should be the same as that used for younger patients, that is, HbA1c ≤7%, while trying to minimize the risk of hypoglycemia.

However, subgroups of patients older than 65 years of age, in which the risk of hypoglycemia is higher, must be taken into account and need adjustment of their treatment goals. Although there are no clinical experiments that quantify this risk, it was decided that patients who are fragile or who suffer from dementia or depression, as well as those receiving end-of-life care, require individualized management. To maintain this focus, it was decided to consider measures developed by other groups that have generated treatment recommendations for these patients based on expert consensus 57- 59. The decision of the group producing the guide was therefore to suggest treatment goals with a maximum HbA1c of 8% in functionally dependent patients, including those who are fragile or demented, in line with the definitions suggested by the IDF 58.

**Recommendations.**

25. In patients with DMT2 who are older than 65 years of age, it is recommended not to intensify treatment to reach HbA1c values close to normal (<6.5% HbA1c).

**Strong recommendation against.** Quality of evidence high. ⴲ ⴲ ⴲ ⴲ

26. In patients with DMT2 who are older than 65 years of age, are functionally independent, and are free of other major comorbidity factors, it is recommended to provide treatment to reach the same HbA1c levels as recommended for younger patients (≤7%). 

**Strong recommendation in favor. **Quality of evidence high. ⴲ ⴲ ⴲ ⴲ

27. In patients with DMT2 who are older than 65 years of age and who exhibit fragility* or dementia**, or who are expected to have a higher-than-usual risk of hypoglycemia***, less intensive treatment is recommended, with target HbA1c values between 7% and 8%.

**Weak recommendation in favor. **Expert consensus.

* Patients with significant fatigue and severe restrictions on mobility or strength, who are at greater risk of falls and institutionalization.

** Patients with a cognitive deficit leading to significant memory problems, disorientation, and personality changes, or who have difficulty taking care of themselves.

*** Patients undergoing treatment with drugs that cause hypoglycemia (sulfonylureas, metiglinides, or insulin) and have other factors that could increase this risk (depression, lack of social support, lack of appetite, or intolerance to ingestion).

28. In patients with DMT2 who are receiving end-of-life care**+**, it is recommended to limit treatment objectives to avoiding symptomatic hyperglycemia.

**Weak recommendation in favor. **Expert consensus**.**

**+** Patients who satisfy the criteria for terminal illness defined in Colombian Law 1733 (2014) or Resolution 1216 (2015) (On the right to die with dignity). A person with a terminal illness is defined as anyone who has a serious illness or pathological condition, which has been precisely diagnosed by an expert physician; which exhibits a progressive and irreversible character; which has a fatal prognosis imminently or in a relatively short period of time; which is not susceptible to a curative treatment of proven efficacy that would change the prognosis of imminent death; or when the therapeutic resources used in an effort to cure have ceased to be effective.

**Good clinical practice guidelines**

✔Considering that the population older than 65 years of age is highly heterogeneous, it is necessary in each case to perform an integrated functional evaluation of the patient to establish therapeutic objectives. 

**Clinical question 8. ¿In adults with DMT2 and cardiovascular disease, is an individualized determination of glycemic control goals needed that is different from the goal of achieving a glycosyladic hemoglobin index equal to or less than 7%?.**

**Answer to question 8. **Various systematic review studies have been identified that compare intensive treatment with glycemic index-reducing drugs and/or insulin with the goal of achieving HbA1c values close to normal by means of conventional treatment schemes with less demanding goals 53,60. These studies included and analyzed independently patients with antecedents of coronary disease. A systematic review 53 has documented that when intensive treatment is compared with conventional treatment, both in patients with antecedents of cardiovascular disease and those having no such antecedents, there is no change in the risk of dying from cardiovascular causes, either in the group with cardiovascular disease (RR= 1.13; CI 95%= 0.81-1.57) or in the group without such disease (RR= 0.89; CI 95%= 0.74-1.08). 

An independent analysis of the ACCORD (Action to Control Cardiovascular Risk in Diabetes) study 60, in which approximately one-third of the subjects with DMT2 had antecedents of established cardiovascular disease, revealed that intensive treatment rather than conventional treatment did not change the risk of suffering the primary outcome. However, the study was discontinued early when a higher mortality rate was observed in the intensive treatment group than in the conventional treatment group. In the pre-established subgroup analysis, no difference in the incidence of mortality was found between those who did or did not have previous cardiovascular disease, although the latter had a significantly lower incidence of primary compound outcomes (defined as infarctions, strokes, or death from cardiovascular causes). 

Similar observations were made in a systematic review of four studies, including the one just mentioned 61. An exploratory subgroup analysis showed that when comparing intensive with conventional therapy, there seemed to be a differential effect on major cardiovascular outcomes in patients with and without antecedents of macrovascular disease, which was significantly favorable to the latter and not significant for the former (HR= 1.00; CI 95%= 0.89-1.13, vs HR= 0.84, CI 95%= 0.74-0.94 respectively; *p*= 0.04 for the interaction). However, in another meta-analysis, the authors found no interaction between antecedents of ischemic heart disease and the effect of intensive glycemic control on cardiovascular events, including fatalities 62. 

In accordance with the evidence presented, it has been concluded that intensifying treatment to reach HbA1c goals close to normal values in patients with antecedents of cardiovascular disease did not show any reduction in the risk of death or in outcomes of cardiovascular origin, including major cardiovascular events. It was also concluded that there was no justification for recommending a more aggressive treatment goal in patients with antecedents of cardiovascular disease. 

**Recommendations.**

29. In patients with DMT2 and with antecedents of cardiovascular disease, the glycosyladic hemoglobin goal *s*hould not be different from the goal for patients in general (less than or equal to 7%), and intensifying treatment to reach an HbA1c value close to normal (HbA1c <6.5%) is not recommended.

**Strong recommendation against. **Quality of evidence moderate. ⴲ ⴲ ⴲ ⴱ

### Topic 5: detection of complications

**Assumptions. **People with DMT2 have a 2 to 4 times greater risk of suffering a coronary event compared to those without diabetes, and a number of epidemiological studies have demonstrated that this risk is equivalent to that of people without diabetes, but with coronary disease. 

Because in this sense they are equivalent to people with coronary disease, people with DMT2 must strictly control cardiovascular risk factors, applying the same kind of strict management that is recommended for secondary prevention of coronary disease by controlling lipids and blood pressure. In applying this treatment, the question arises of screening for coronary disease in asymptomatic patients. Screening tests for coronary disease include the conventional stress test, pharmacological stress tests with echocardiography or nuclear medicine perfusion images, and quantification of coronary calcium by means of computerized tomography. Electrocardiograms are routinely taken in those over 40 years of age, but they are not considered sufficient to screen for coronary disease because of their low sensitivity and specificity.

The vascular damage produced by sustained hyperglycemia in patients with DMT2 also appears in the capillaries as microangiopathy. The principal clinical manifestations occur in the retina (retinopathy), the glomerules (nephropathy), and the peripheral nerves (neuropathy).

Because DMT2 tends to begin without symptoms, much time may elapse between the start of the disease and its diagnosis, and it is not uncommon to find clinical manifestations of microangiopathy in patients with recently diagnosed DMT2. This imposes an obligation for systematic search (screening) for these complications at the beginning and every year afterwards, with the aim of intensifying control to avoid progression of the disease. Screening for retinopathy is performed by photography of the non-dilated retina or by ophthalmoscopy performed by a specialized professional. Screening for nephropathy is done by measuring the amount of albumin in the urine and estimating the glomerular filtration rate. Screening for neuropathy is done by evaluating sensitivity to vibration and touch in the ankles and feet.

**Clinical question 9. ¿Should adults with DMT2 and without symptoms of coronary insufficiency be screened for coronary artery disease?.**

**Answer to question 9. **After reviewing the best evidence available, three clinical studies were found 63- 65 and evaluated. It was concluded that screening for silent coronary disease in patients with asymptomatic type 2 diabetes with or without cardiovascular risk factors did not reduce the incidence of cardiovascular events: it had no impact on acute nonfatal myocardial infarctions (RR= 0.61; CI 95%= 0.29-1.29), or on death from all causes (RR= 1.18; CI 95%= 0.72-1.93). Moreover, it had no statistically or clinically significant impact on the frequency of revascularization, which was similar in the controlled clinical studies. 

Finally, it is worth mentioning that performing these tests on asymptomatic DMT2 patients leads to unnecessary and costly procedures, which are eventually carried out on unscreened patients when they present symptoms, thus ending up with the same burden of illness.

**Recommendations.**

30. In patients with DMT2 without symptoms of coronary insufficiency, it is suggested not to perform screening for coronary artery disease. 

**Weak recommendation against. **Quality of evidence low. ⴲ ⴱ ⴱ ⴱ

**Good clinical practice guidelines**.

✔In all patients with DMT2, cardiovascular risk factors must be managed as if dealing with a person with established coronary disease, with the one important exception being administration of low-dose aspirin, which is not recommended for patients with DMT2 without established cardiovascular disease.

✔The diagnostic procedure for coronary disease should be initiated once the patient presents symptoms suggestive of coronary disease, and according to the findings, opportune and appropriate treatment should be undertaken.

✔The person with DMT2 must know the classic symptoms of coronary ischemia as well as less common ones like sudden difficulty in breathing, so that he/she can identify them quickly. 

**Clinical question 10. ¿In adults with DMT2, how should the results of screening for albuminuria affect treatment?.**

**Answer to question 10. **A systematic review of efforts to optimize screening for renal disease in diabetics and the impact of treatment 66 demonstrated in the case of DMT2 that urinary excretion of albumin decreased by 21% (CI 95%= 7%-32%) in those with normoalbuminuria and by 27% (CI 95%= 15%-38%) in those with microalbuminuria after treatment with Angiotensin-converting enzyme inhibitors (ACEI) or with angiotensin receptor blockers (ARB II) compared with those who did not receive this treatment, although the response was highly varied (I^2^= 85% and 87% respectively). Both the patients with normoalbuminuria and those with microalbuminuria benefited from these treatments in terms of progression and regression. The relative risk of progressing from normo- to microalbuminuria was 0.84 (CI 95%= 0.79-0.89), and that from micro- to macroalbuminuria was 0.52 (CI 95%= 0.43-0.63). Moreover, a larger number of patients regressed from micro- to normoalbuminuria in the treatment group (RR= 1.2; CI 95%= 1.12-1.29), although the heterogeneity was high (75%). There was no significant effect on glomerular filtration or on mortality, although the follow-up period was very short to evaluate this type of outcome. No difference in the response was found after performing a sensitivity analysis between use of hypertensive and nonhypertensive controls (*p*= 0.86).

In DMT2 patients with normal blood pressure, treatment with ARA II also reduced urinary excretion of albumin (relation of means= 0.57; CI 95%= 0.47-0.69) 67. A similar effect can be hoped for with use of ACEI 68.

However, the evidence for microalbuminuria treatment in DMT2 patients with normal blood pressure is not sufficient to demonstrate an effect on cardiovascular events or outcomes that is related to renal function 69- 71. In addition, it should be taken into account that in the meeting of the Food and Drug Administration (FDA) and the National Kidney Foundation in 2008, it was concluded that the available evidence is insufficient to determine that albuminurea levels can be used as a clinical diagnostic measure in patients with diabetes.

No evidence was found in the documents previously presented regarding the following significant outcomes: cardiovascular disease, progression to stage III nephropathy, or the need for dialysis. 

**Figure 5.**
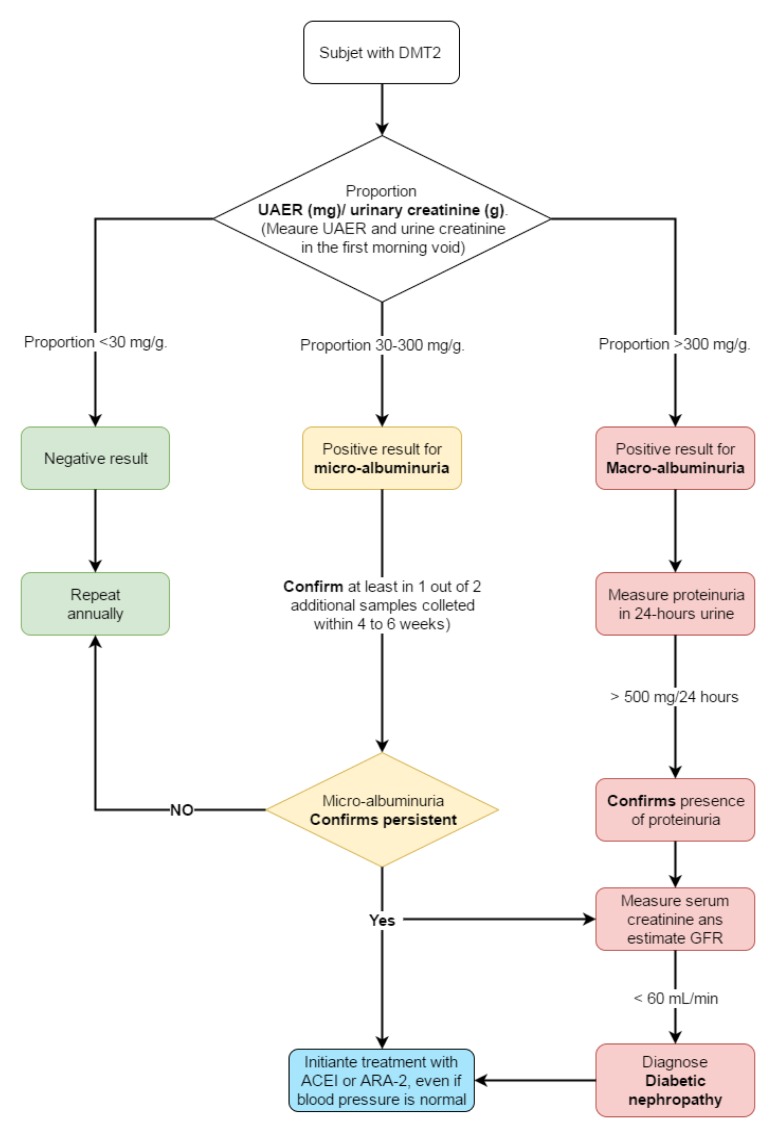
Screening for urinary albumin excretion rate. UAER: urinary albumin excretion rate, GFR: glomerular filtration rate, ACEI: Angiotensin-converting enzyme inhibitors

**Recommendations** (Fig. 5).

31. In patients with DMT2, it is suggested to begin treatment with ACEI or ARB II when persistent microalbuminuria is detected*, and although the patient does not have arterial hypertension. 

**Weak recommendation in favor. **Quality of evidence moderate. ⴲ ⴱ ⴱ ⴱ

32. It is recommended *not to give* treatment with IECA or ARA2 to patients with DMT2 who do not have arterial hypertension or microalbuminuria*. 

**Weak recommendation against. **Quality of evidence moderate. ⴲ ⴱ ⴱ ⴱ

* Proportion ≥30 mg of albuminuria/g of creatinuria in a sample taken from the first urine of the morning, or ≥30 mg albumin in urine collected over 24 hours, which persists in a second sample taken after 4 to 6 weeks.

**Good clinical practice guidelines**.

✔To establish the presence of microalbuminuria, it is preferable to measure albuminuria and creatinuria in a sample from the first urine of the morning and to calculate the ratio microalbuminuria (mg)/creatinuria (g). 

✔The screening test for microalbuminuria must be performed annually starting with DMT2 diagnosis. Because multiple factors can increase albumin excretion over the short term, persistence must be confirmed by at least 2 or 3 measurements on samples taken at intervals of 4 to 6 weeks.

✔Diabetic nephropathy is diagnosed when, in addition to the presence of microalbuminuria, the estimated glomerular filtration rate (GFRe) is lower than 60 mL/min. GFRe is calculated using formulas such as MDRD, Cockroft, or CKD-Epi that take into account serum creatinine, age, and sometimes weight. They are also adjusted for gender and racial differences. The last formula is presently the most widely used.

✔In patients with DMT2, strict control of arterial hypertension will prevent or reduce the progression of diabetic nephropathy. Treatment is begun with ACEI or ARA2, but other antihypertensives may be added as necessary to reach a systolic arterial pressure goal between 130 and 139 mmHg and a diastolic arterial pressure goal ≤80 mmHg.

✔When albuminuria is >300 mg/g creatinine (>300 mg in 24 hours), it is classified as macroalbuminuria and is considered equivalent to the presence of proteinuria. Its persistence indicates a major risk of mortality and a progression to advanced stages of kidney failure and requires even stricter management.

## Follow-up and implementation indicators for the Good Clinical Practice 

The proposed indicators are designed to support the implementation process, evaluate adherence to the recommendations, and validate the impact of the Good Clinical Practice (Table 3). Provided that the information obtained is timely, trustworthy, and accurate, these indicators can be used as inputs to be fed back into the implementation process and encourage development of the Good Clinical Practice. The full development process for these indicators is described in the complete version of the guide, which is available (in Spanish) on the Web site of the Colombian Ministry of Health and Social Welfare (http://gpc.minsalud.gov.co/guias/Documents/ diabetes/DIABETES_TIPO_2_COMPLETA.pdf). 

**Table 3.** Follow-up indicators for the implementation of the guidelines

Type of indicator	Name of indicator	Operational formulation	Frequency	Primary source	Goal
structural	Percentage of laboratories that measure HbA1c in the manner recommended by the NGSPP (www.ngsp.org).	Number of laboratories measuring HbA1c in the manner recommended by the NGSPP / Number of laboratories measuring HbA1c × 100	Annual	EPS	First year: 50%, Second year: 100%
Process	Percentage of patients with T2DM (Code CIE-10: E11) who undergo at least two HbA1c measurements per year	(Number of patients with a clinical diagnosis of T2DM [Code CIE10: E11] who undergo at least two HbA1c measurements per year) / (Number of patients with a clinical diagnosis of T2DM (Code CIE10: E11)) * 100	Annual	IPS	First year: 60% Second year: 90%
	Percentage of patients with T2DM (Code CIE10: E11) who undergo an annual urinary albumin excretion rate (UAER) test	(Number of patients with a clinical diagnosis of T2DM (Code CIE10: E11) who undergo an UAER test during the year) / (Total number of patients with a clinical diagnosis of T2DM (Code CIE10: E11)) * 100	Annual	IPS	Primer año: 50%. Tercer año: 90%
Resultado	Percentage of patients with T2DM [Code CIE10: E11] with glycosylated hemoglobin (HbA1c) levels less than or equal to 7%	(Number of patients with a clinical diagnosis of T2DM (Code CIE10: E11) with HbA1c values ≤7%) / (Total number of patients with a clinical diagnosis of T2DM (Code CIE10: E11)) * 100	Every 6 months	IPS	First year: 40% Second year: 70%

EPS= Health Promotion Organizations, IPS= Health Provider Institutions

## Electronic supplementary material

Table s1. FINDRISC screening test for diabetes and other glucose regulation abnormalities .
