# Supplementary material for: Clinical practice guideline for the prevention, early detection, diagnosis, management and follow up of type 2 diabetes mellitus in adults
Source: Colomb Med (Cali). 2016 Jun 30;47(2):109–31. (PMC4975132)
Supplement: Supplementary file 1 [file 1657-9534-cm-47-02-00109-s1.pdf]

Supplementary material

Table 1s. FINDRISC screening test for diabetes and other glucose regulation abnormalities

Nombre ______________________ ID/Contacto ______________

1. Edad _________ años cumplidos

❑ Menos de 45 años (0 p.)
❑ 45-54 años (2 p.)
❑ 55-64 años (3 p.)
❑ Más de 64 años (4 p.)

2. Índice de masa corporal: Peso ____ kg, Talla _____ mts, IMC: _____ kg/m²

(Se calcula dividiendo el peso en kilos) por la talla en metros elevada al cuadrado.
❑ Menor de 25 kg/m² (0 p.)
❑ Entre 25-30 kg/ m² (1 p.)
❑ Mayor de 30 kg/ m² (3 p.)

3. Perímetro de cintura __________ cm

Pasar el metro en medio del reborde costal y la cresta ilíaca de ambos lados y totalmente horizontal (aunque no pase por el ombligo). La persona debe respirar normalmente un par de veces y se toma la medida menor (cuando exhale el aire)

Hombres ❑ Menos de 94 cm (0 p.) ❑ Igual o mayor a 94 cm. (4 p.)
Mujeres ❑ Menos de 90 cm (0 p.) ❑ Igual o mayor a 90 cm. (4 p.)

4. ¿Realiza habitualmente (a diario) al menos 30 minutos de actividad física, en el trabajo y/o en el tiempo libre?:

❑ Sí (0 p.) ❑ No (2 p.)

5. ¿Con qué frecuencia come verduras o frutas?:

❑ Todos los días (0 p.)
❑ No todos los días (1 p.)

6. ¿Toma medicación para la hipertensión regularmente?:

❑ No (0 p.) ❑ Sí (2 p.)

7. ¿Le han encontrado alguna vez valores de glucosa altos?

(Ej. en un control médico, durante una enfermedad, durante el embarazo)
❑ No (0 p.) ❑ Sí (5 p.)

8. ¿Se le ha diagnosticado diabetes (tipo 1 o tipo 2) a alguno de sus familiares?

(Nota: La diabetes "por edad" o "ya de viejo" también cuenta)
❑ Sí: padres, hermanos o hijos (5 p.)
❑ Sí: abuelos, tíos, primos hermanos (3 p.)
❑ Otros parientes o ninguno (0 p.)

Puntaje Total (máximo 26 p.)

Interpretación del puntaje: Igual o mayor a 12 = Alta probabilidad de tener Diabetes u otra anormalidad de la regulación de la glucosa
